# Conical islands of TiO_2_ nanotube arrays in the photoelectrode of dye-sensitized solar cells

**DOI:** 10.1186/s11671-015-0737-2

**Published:** 2015-02-11

**Authors:** Woong-Rae Kim, Hun Park, Won-Youl Choi

**Affiliations:** Department of Metal and Materials Engineering, Gangneung-wonju National University, Gangneung, 210-720 South Korea; Korea Institute of Science and Technology Information, Seoul, 130-741 South Korea; Research Institute for Dental Engineering, Gangneung-Wonju National University, Gangneung, 210-720 South Korea

**Keywords:** Dye-sensitized solar cells, TiO_2_ nanotube, Conical islands, Anodic oxidation

## Abstract

Ti conical island structures were fabricated using photolithography and the reactive ion etching method. The resulting conical island structures were anodized in ethylene glycol solution containing 0.25 wt% NH_4_F and 2 vol% H_2_O, and conical islands composed of TiO_2_ nanotubes were successfully formed on the Ti foils. The conical islands composed of TiO_2_ nanotubes were employed in photoelectrodes for dye-sensitized solar cells (DSCs). DSC photoelectrodes based on planar Ti structures covered with TiO_2_ nanotubes were also fabricated as a reference. The short-circuit current (*J*_sc_) and efficiency of DSCs based on the conical island structures were higher than those of the reference samples. The efficiency of DSCs based on the conical island structures reached up to 1.866%. From electrochemical impedance spectroscopy and open-circuit voltage (*V*_oc_) decay measurements, DSCs based on the conical island structures exhibited a lower charge transfer resistance at the counter cathode and a longer electron lifetime at the interface of the photoelectrode and electrolyte compared to the reference samples. The conical island structure was very effective at improving performances of DSCs based on TiO_2_ nanotubes.

**Electronic supplementary material:**

The online version of this article (doi:10.1186/s11671-015-0737-2) contains supplementary material, which is available to authorized users.

**Graphical Abstract:**
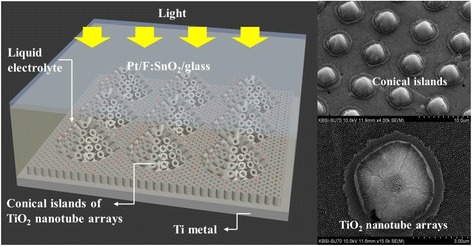
Conical islands of TiO_2_ nanotube arrays are fabricated by an anodizing process with Ti protruding dots which have a conical shape. The conical islands are applied for use in DSC photoelectrodes. DSCs based on the conical islands of TiO_2_ nanotube arrays have the potential to achieve higher efficiency levels compared to DSCs based on normal TiO_2_ nanotubes and TiO_2_ nanoparticles because the conical islands of TiO_2_ nanotube arrays enlarge the surface area for dye adsorption.

## Background

Dye-sensitized solar cells (DSCs) have received great interest as promising alternatives to conventional silicon solar cells [1]. Their low cost and easy fabrication processes are powerful advantages. For example, DSCs can be fabricated without the high-cost vacuum equipment required for Si cells, such as plasma-enhanced chemical vapor deposition (PECVD) and atomic layer deposition (ALD). Wet etching processes, such as saw damage etching and texturing, which are widely used in the fabrication of Si solar cells, are not required in DSCs, which simplifies their fabrication.

Gratzel and O’Regan achieved remarkably high-efficiency DSCs by using mesoporous TiO_2_ nanoparticle-based films [1]. Recently, Burschka et al. reported high efficiency, up to 15%, by fabricating inorganic-organic hybrid solar cells containing perovskite compounds [2]. However, the efficiencies of DSCs are still too low for commercialization compared with conventional Si solar cells.

The main components which affect the performance of DSCs are the dyes [3-13], photoelectrodes [14-38], counter cathodes [39-46], and electrolytes [47-51]. Among these components, increasing the surface area of the photoelectrodes and reducing charge recombination between the photoelectrodes and electrolytes are critical factors in improving the efficiencies of DSCs.

TiO_2_ nanoparticle structures have been widely used for the photoelectrodes of DSCs. However, structural defects which exist at the contacts between the TiO_2_ nanoparticles not only hinder electronic diffusion across TiO_2_ nanocrystalline films but also act as charge recombination sites [20-22,24,26]. To address this issue, TiO_2_ nanostructures such as nanowires [31,33,34], nanotubes [20-30,37,38,52,53], nanohemispheres [35,36], and nanoforests [32] have been applied to the photoelectrodes of DSCs as alternatives to the conventional TiO_2_ nanoparticle structure.

Of these nanostructures, the TiO_2_ nanotube has received a great deal of attention due to its one-dimensional charge transport. Charge percolation in TiO_2_ nanotube-based films is easier than that in TiO_2_ nanoparticle-based films [20-22]. It has been reported that electron lifetimes in TiO_2_ nanotube-based films are longer than those in TiO_2_ nanoparticle-based films [26].

In this study, conical island shapes were patterned on a Ti substrate to increase the specific surface area for dye adsorption. Ti surfaces with these conical islands were anodized, and TiO_2_ nanotube arrays were formed on the entire Ti surface. Conical islands composed of TiO_2_ nanotubes were successfully fabricated and applied as the photoelectrodes of DSCs. Planar Ti photoelectrodes were also prepared with TiO_2_ nanotube arrays, and then the characteristics and performance of the conical island photoelectrode structure were compared with those of the planar photoelectrode structure. To our knowledge, this is the first trial to report the fabrication of conical islands composed of TiO_2_ nanotubes and their application to DSCs.

## Methods

The experimental procedures for making conical islands composed of TiO_2_ nanotubes are very similar to those found in our previous report [30]. To make conical islands on the surface of 0.5-mm-thick Ti foils (99%, Alfa Aesar Co., Ward Hill, MA, USA), 5-μm-thick photoresists (PR; L-300, Dongjin Co., Hwaseong-si, South Korea) were coated on the surface of Ti foils using a spin-coater (Mark-8 Track, TEL Co., Tokyo, Japan). The spin-coated photoresists were softly baked at 120°C for 120 s and hardly baked at 110°C for 5 min. A dot-patterned photomask was used to make PR patterning by UV light exposure. The UV light, which had an energy of 14.5 mJ/s, was illuminated for 5 s, and the PR was developed. The PR in the UV-unexposed area was removed.

The PR-patterned Ti foil was dry-etched at 25°C for 40 min by using reactive ion etching (RIE) equipment (ICP380, Oxford Co., Abingdon, Oxfordshire, UK). BCl_3_ and Cl_2_ gases were used in the RIE process with a top power of 800 W and a bottom power of 150 W. Photoresists on the UV-exposed area played a role in protecting the flat Ti surface during the RIE process. Only the Ti surface in the UV-unexposed area was etched out. The remaining photoresists after the RIE process were stripped at 250°C for 20 min using a photoresist stripper (TS-200, PSK Co., Hwaseong-si, South Korea). O_2_ and N_2_ gases were used for photoresist stripping with a power of 2,500 W.

**Figure 1:**
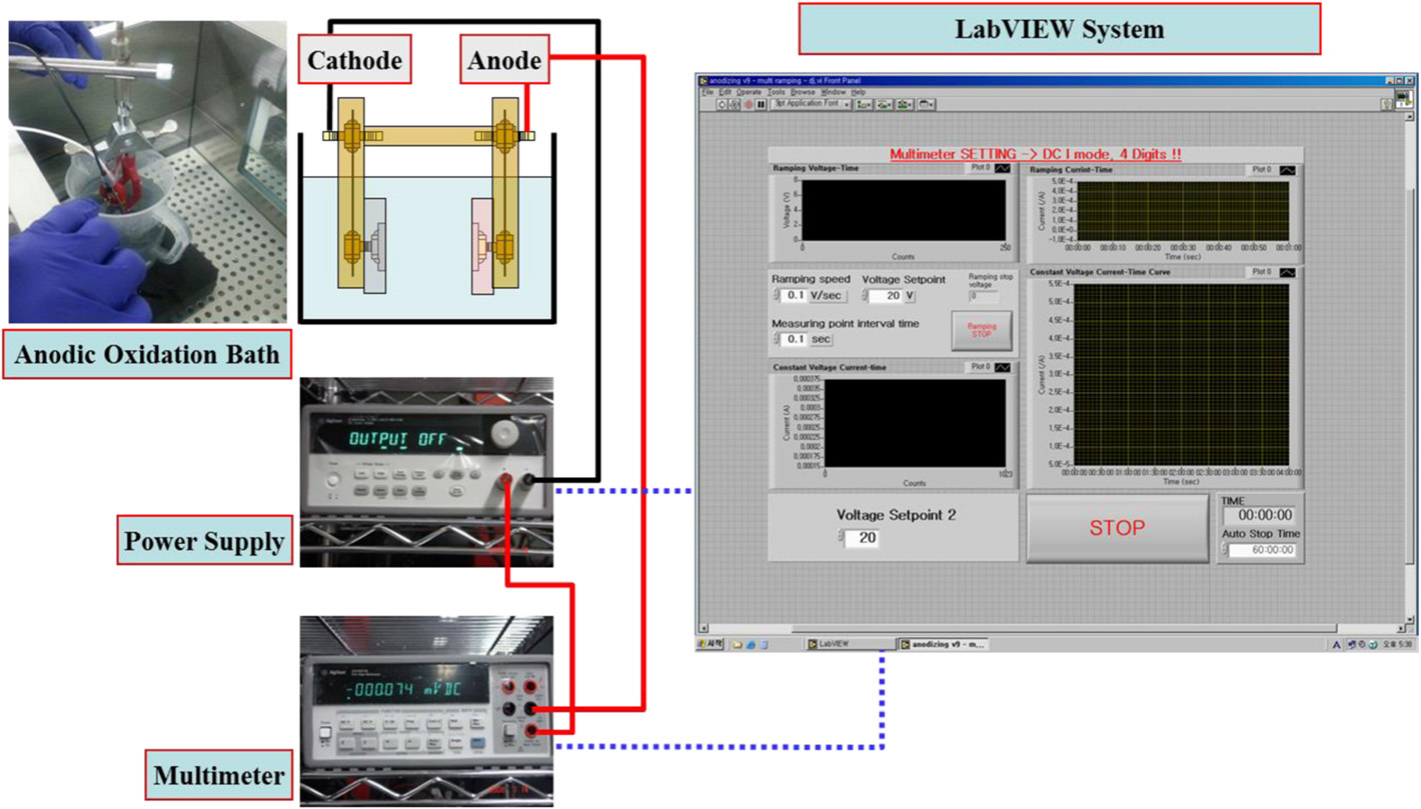
**Schematic diagram of the anodic oxidation system.**

Before the anodizing process, the Ti foils patterned with conical islands were successively ultrasonicated with acetone, ethanol, and deionized (DI) water to remove any residues on the surface of the Ti foils. Conical islands composed of TiO_2_ nanotubes were fabricated by anodic oxidation of the patterned Ti foils in ethylene glycol solution. The schematic diagram of the anodic oxidation system is shown in Figure 1.

To obtain a proper microstructure of TiO_2_ nanotube arrays, anodic oxidation voltages and times applied by a DC power supply were computer-controlled by a LabVIEW program. The ethylene glycol solution included 0.5 wt% NH_4_F and 0.2 vol% H_2_O. A constant potential of 60 V with a ramping speed of 1 V/s was applied between the anode and the cathode. Pt metal was used as a counter cathode. Anodizing time was varied to control the optimum thickness of TiO_2_ nanotubes in the conical islands. As-anodized TiO_2_ nanotubes were rinsed with DI water and annealed at 500°C for 1 h. The morphologies of the conical islands composed of TiO_2_ nanotubes were studied by field emission scanning electron microscopy (FESEM; Hitachi SU-70, Hitachi, Ltd., Chiyoda-ku, Japan) at Korea Basic Science Institute. As-anodized and annealed TiO_2_ nanotubes were analyzed by X-ray diffraction (XRD; Rigaku D/MAX-RC, Shibuya-ku, Japan, Cu Kα radiation) to confirm their crystallization.

The Ti substrates with conical islands composed of TiO_2_ nanotubes were then prepared for use as the photoelectrodes of DSCs. The TiO_2_ photoelectrodes were immersed at room temperature for approximately 1 day in an ethanol solution containing 3 × 10^−4^ M cis-bis(isothiocyanato)bis(2,2′bipyridyl-4,4′dicarboxylato) ruthenium(II) bis-tetrabutylammonium (N719) dye. The dye-adsorbed photoelectrodes were rinsed with ethanol solution and were dried at room temperature. Pt-coated fluorine-doped tin oxide (FTO) glasses were prepared as counter electrodes by spin coating 0.7 mM H_2_PtCl_6_ solution in 2-propanol at 500 rpm for 10 s and subsequently annealing at 380°C for 30 min. The dye-adsorbed photoelectrodes and Pt-coated FTO glasses were spaced by using 60-μm Surlyn film purchased from DuPont Co., Ltd. (Wilmington, DE, USA). The liquid electrolyte was prepared by dissolving 0.6 M 1-hexyl-2,3-dimethylimidazolium iodide (C6DMIm), 0.05 M iodine, 0.1 M lithium iodide, and 0.5 M 4-tert-butylpyridine in 3-methoxyacetonitrile. Current-voltage (*J*-*V*) characteristics were measured under AM 1.5G condition (Keithley Model 2400 source measure unit, Keithley Instruments, Inc., Cleveland, OH, USA). Electrochemical impedance spectroscopy (EIS) behaviors were analyzed at open-circuit condition and in the frequency range of 100 kHz to 100 mHz. A 1,000-W xenon lamp (Oriel, 91193, Newport Co., Stratford, CT, USA) was used as a light source for one sun condition.

## Results and discussion

**Figure 2:**
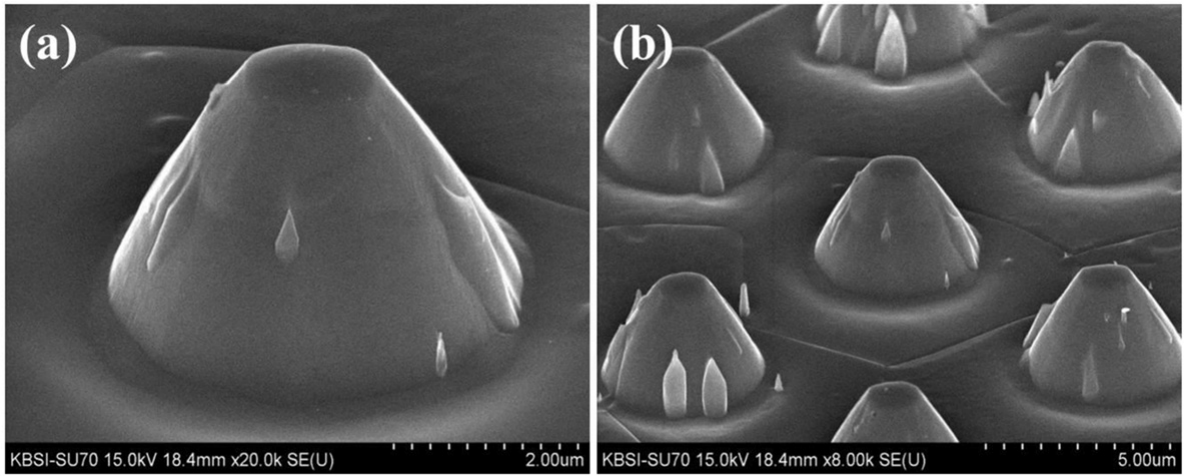
**FESEM images of the conical island structure of the Ti surface fabricated by dry etching.** At magnifications of **(a)** × 20,000 and **(b)** × 8,000.

Figure 2 shows the FESEM images of the conical islands formed on the Ti surface by the RIE method. In our previous report, we fabricated arrays of protruding Ti cylinders [30]. By increasing the etching time, the morphology of the Ti surface can be converted from protruding cylinders to conical islands, as shown in Figure 2. In this process, negative photoresists were first spin-coated on the Ti metal surface. Usually, when photoresists are exposed to UV light, they are cross-linked and so block reactive etching ions during the RIE process. The cross-linked photoresist layer protects the Ti surface from physical and chemical etching.

However, photoresists have weaker cross-linking at an edge than at the center. At edges, cross-linked photoresists can be easily damaged during the RIE process if the etching time is long enough. In that case, the cross-linked photoresists at the edge areas can be crushed by the reactive ions after the formation of the Ti protruded cylinders. This allows the protruded cylinders to be converted into conical islands by increasing the dry etching time, as shown in Figure 2a.

The top and the bottom of the produced conical islands have diameters of approximately 1 μm and approximately 4 μm, respectively. The height of the conical islands is approximately 5 μm. Ti conical island arrays can be seen in Figure 2b. The distance between adjacent conical islands is approximately 8 μm and coincides with the photomask patterns.

**Figure 3:**
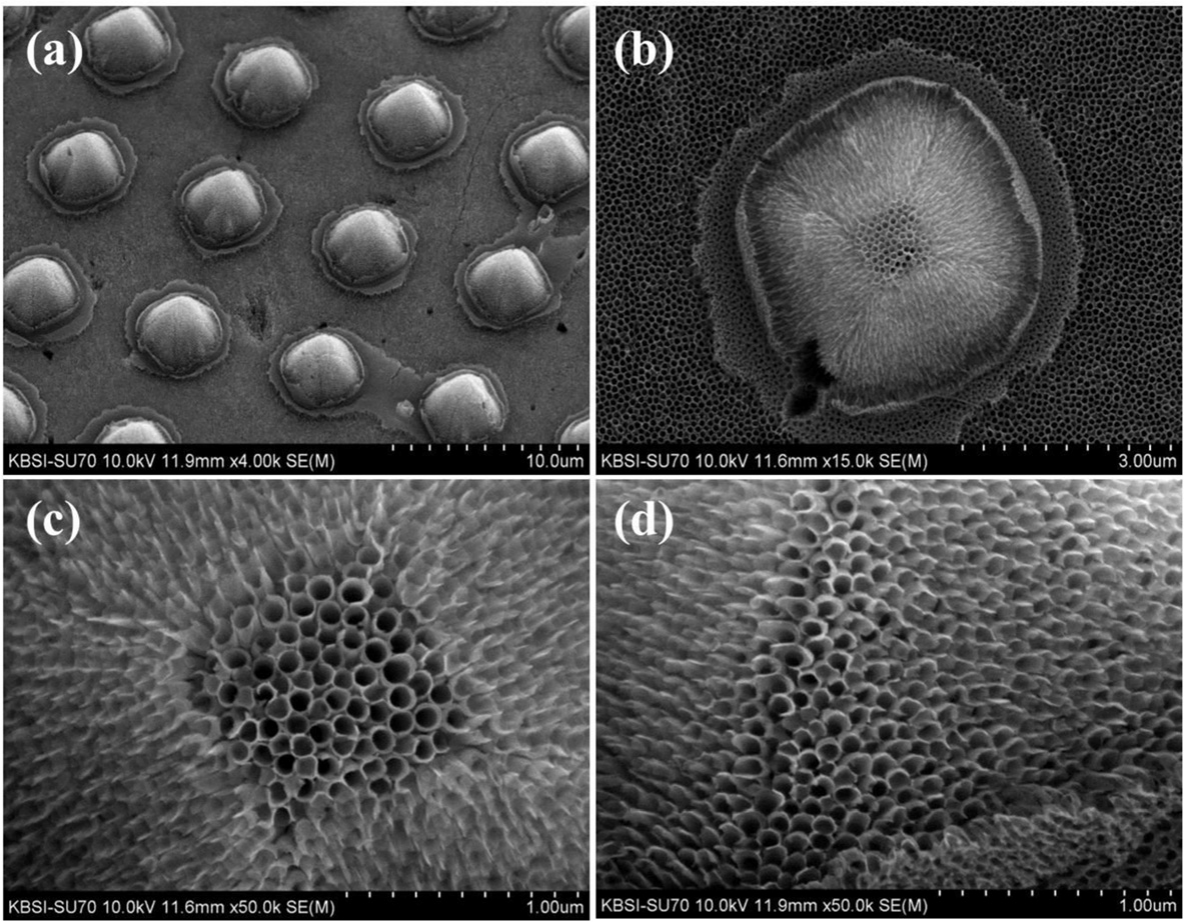
**FESEM images of the conical island structure composed of TiO**_**2**_ **nanotube arrays grown by anodic oxidation.** At a constant voltage of 60 V in 0.5 wt% NH_4_F- and 0.2 vol% H_2_O-containing ethylene glycol solution; at magnifications of **(a)** × 4,000, **(b)** × 15,000, **(c)** × 50,000, and **(d)** × 50,000.

Figure 3 shows FESEM images of the conical islands composed of TiO_2_ nanotubes. The TiO_2_ nanotubes were grown by anodic oxidation of Ti metal in 0.5 wt% NH_4_F- and 0.2 vol% H_2_O-containing ethylene glycol solution. A constant voltage of 60 V with a ramping speed of 1 V/s was applied between the cathode and the anode during anodic oxidation. Fluoride ions in the ethylene glycol solution can anisotropically etch Ti and Ti oxide due to the voltage-biased direction. As a result, highly ordered TiO_2_ nanotubes are vertically grown on the whole Ti surface, including the conical islands. The inclined planes of the conical islands also have vertically grown TiO_2_ nanotubes on their surfaces. The underlying mechanism is discussed in a previous report [30]. The diameter and wall thickness of the fabricated TiO_2_ nanotubes are approximately 80 nm and approximately 20 nm, respectively. The length of the TiO_2_ nanotube arrays was controlled by the anodizing time and was in the range of 1 to 2 μm.

**Figure 4:**
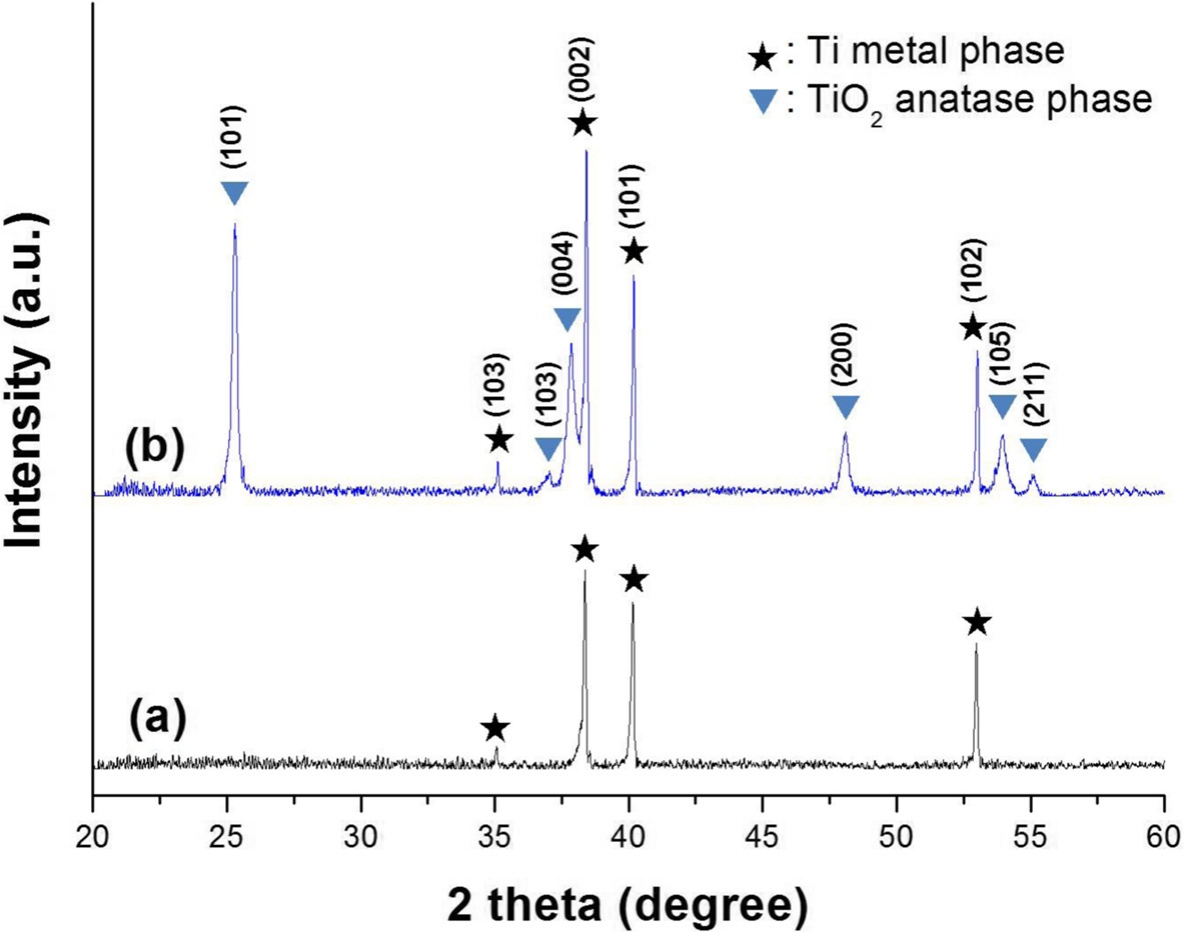
**X-ray diffraction patterns of the (a) as-anodized and (b) annealed conical island structures composed of TiO**_**2**_ **nanotube arrays.**

Figure 4 shows the X-ray diffraction patterns of the as-anodized and annealed conical island structures composed of TiO_2_ nanotube arrays on Ti substrates. The as-anodized conical island structure composed of TiO_2_ nanotube arrays, as shown in Figure 4a, has only Ti metal peaks, and there are no crystalline peaks of Ti oxides. The as-anodized conical island structure can be confirmed as amorphous phase. After annealing at 500°C for 1 h, the anatase peaks of TiO_2_ can be found, as seen in Figure 4b. This reveals that the nanotubes on the conical islands were converted to the anatase phase by the annealing process. The anatase phase has previously shown the best performance when applied in DSCs because the anatase (101) plane provides preferential sites for dye adsorption [54,55].

**Figure 5:**
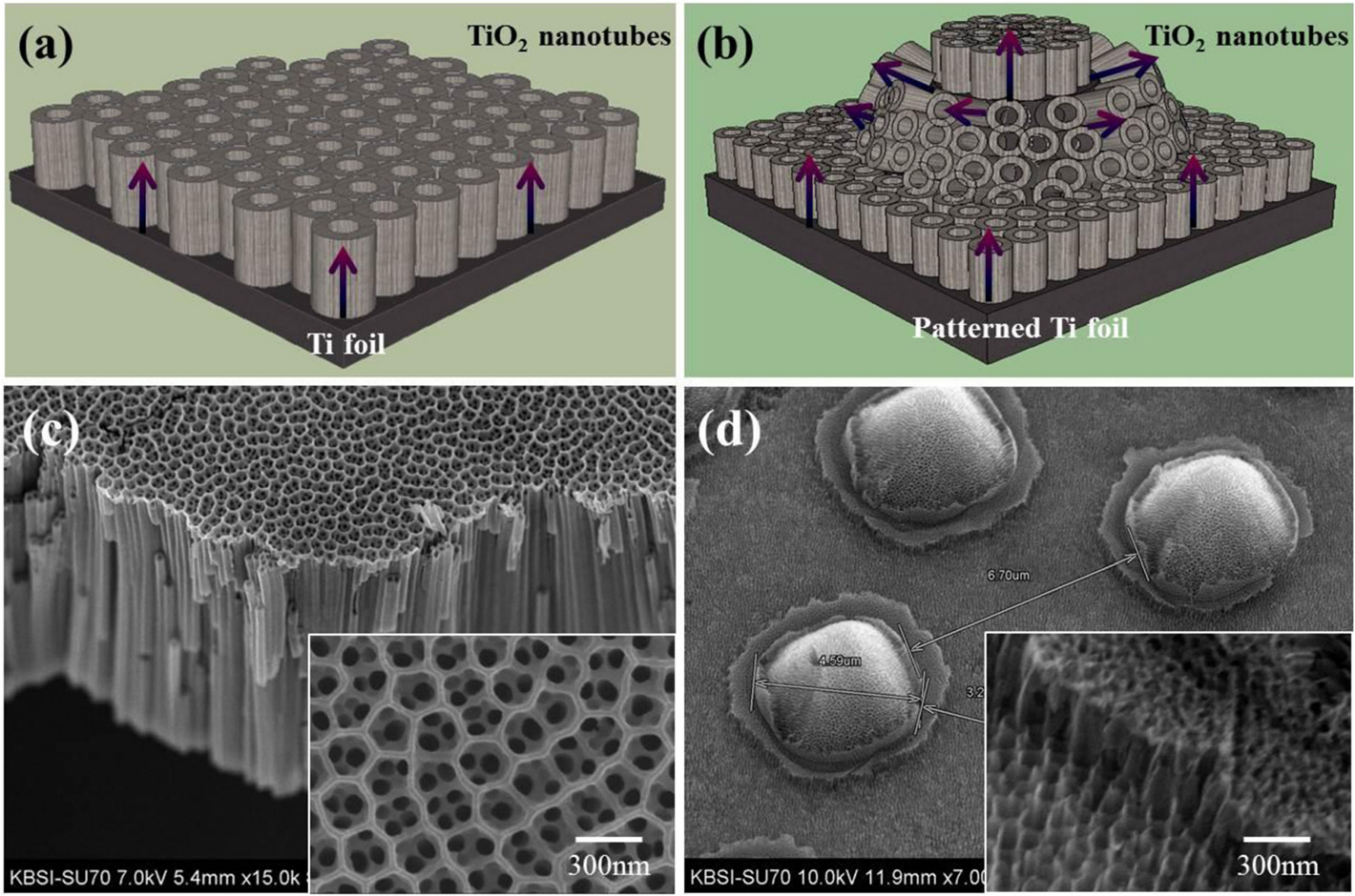
**Illustrations of the photoelectrodes in DSCs and FESEM images.** Illustrations of the photoelectrodes in DSCs with the **(a)** planar structure of TiO_2_ nanotube arrays and **(b)** conical island structure of TiO_2_ nanotube arrays, and FESEM images of the **(c)** planar structure of TiO_2_ nanotube arrays and **(d)** conical island structure of TiO_2_ nanotube arrays.

Figure 5 shows the schematic diagrams and FESEM images of the DSC photoelectrodes prepared with a planar structure and with a conical island structure [30]. The photoelectrode with the planar TiO_2_ nanotube array was prepared by the anodic oxidation of flat Ti foils. On the other hand, the photoelectrode with the conical island structure of TiO_2_ nanotube arrays was fabricated by anodic oxidation of Ti foils which were patterned, etched, and finally shaped like conical islands.

For experimental purposes, both types of TiO_2_ arrays were prepared with nanotube lengths of 1.0, 1.5, and 2.0 μm. Both types of photoelectrodes were fabricated with the identical active cell size of 5 mm × 5 mm. The photoelectrode with the planar TiO_2_ nanotube arrays was later used as a reference sample to compare *J*-*V* characteristics and EIS behaviors with the conical island structure.

**Figure 6:**
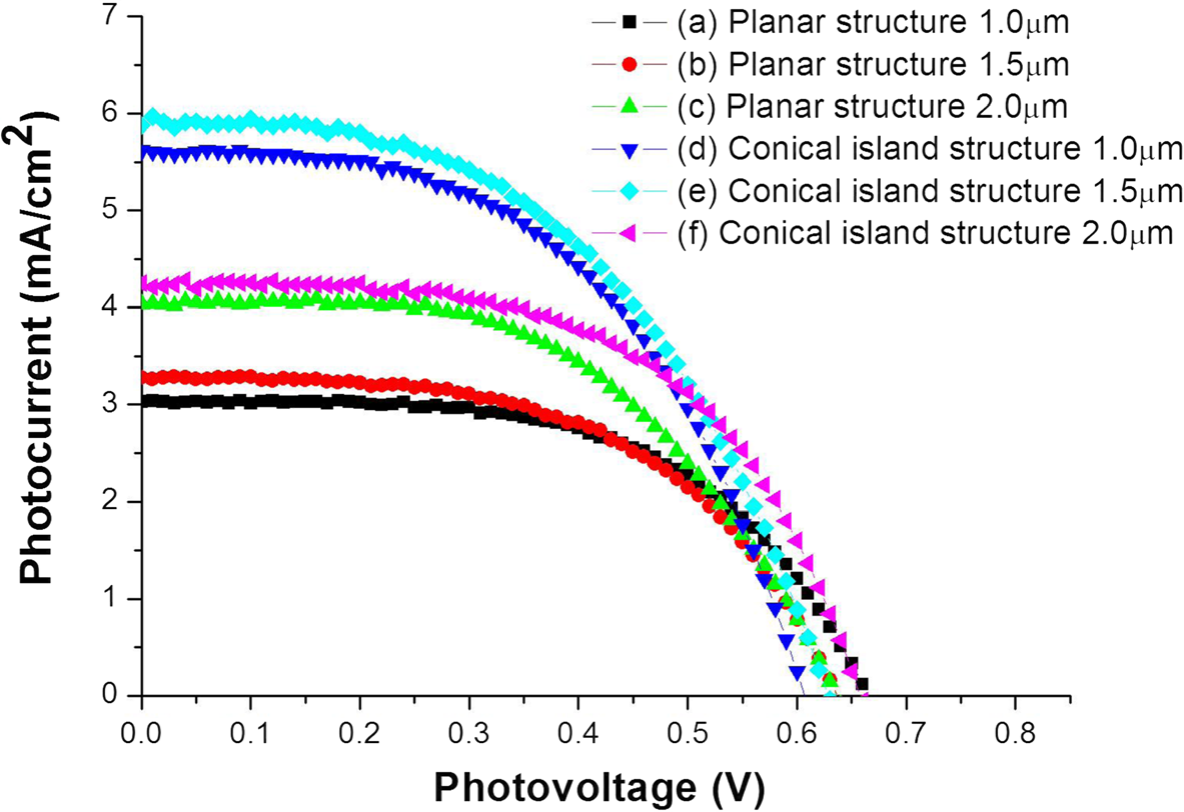
***J*** **-** ***V*** **curves of DSCs based on planar and conical island structures of TiO**_**2**_ **nanotube arrays.** (a) Planar structure, 1.0 μm (average length of TiO_2_ nanotube arrays); (b) planar structure, 1.5 μm; (c) planar structure, 2.0 μm; (d) conical island structure, 1.0 μm; (e) conical island structure, 1.5 μm; and (f) conical island structure, 2.0 μm.

Figure 6 and Table 1 show the *J*-*V* characteristic performances of DSCs based on planar and conical island structures of TiO_2_ nanotube arrays, with different lengths of TiO_2_ nanotubes. The short-circuit current (*J*_sc_) in the planar structure increased with the length of the TiO_2_ nanotube arrays, and such data has been previously reported [30]. Longer TiO_2_ nanotubes provide a larger specific surface area, which adsorbs more dye and produces more photoelectrons.

**Table 1:** ***J*** **-** ***V*** **characteristics of DSCs based on planar and conical island structures of TiO**_**2**_ **nanotube arrays**

**Sample**	**Photoelectrode**	***J***_**sc**_ **(mA/cm** ^**2**^ **)**	***V***_**oc**_ **(V)**	**Fill factor**	**Efficiency**
(a)	Planar structure, 1.0 μm	3.031	0.665	0.574	1.159 ± 0.104
(b)	Planar structure, 1.5 μm	3.279	0.636	0.549	1.147 ± 0.167
(c)	Planar structure, 2.0 μm	4.030	0.636	0.536	1.378 ± 0.092
(d)	Conical island structure, 1.0 μm	4.249	0.658	0.571	1.598 ± 0.067
(e)	Conical island structure, 1.5 μm	5.620	0.606	0.520	1.774 ± 0.21
(f)	Conical island structure, 2.0 μm	5.877	0.628	0.500	1.866 ± 0.279

For the same length of TiO_2_ nanotubes, DSCs based on the conical island structure show higher *J*_sc_ than DSCs based on the planar structure. The added TiO_2_ nanotube arrays on the inclined surfaces of the conical island further increase the surface area available to adsorb the dyes and produce photoelectrons.

DSCs having 2.0-μm-long TiO_2_ nanotube arrays showed the best performances for both planar and conical island structures. The short-circuit current and efficiency for the conical island structures and 2.0-μm-long TiO_2_ nanotubes were 5.877 mA/cm^2^ and 1.866%, respectively. In all cases, the efficiencies of the conical island structure DSCs were higher than those based on the planar structure. The conical island structure was very effective at improving DSC power conversion efficiency. It is also anticipated that conical island structures with TiO_2_ nanotube arrays longer than 2.0 μm will have still higher efficiency.

**Figure 7:**
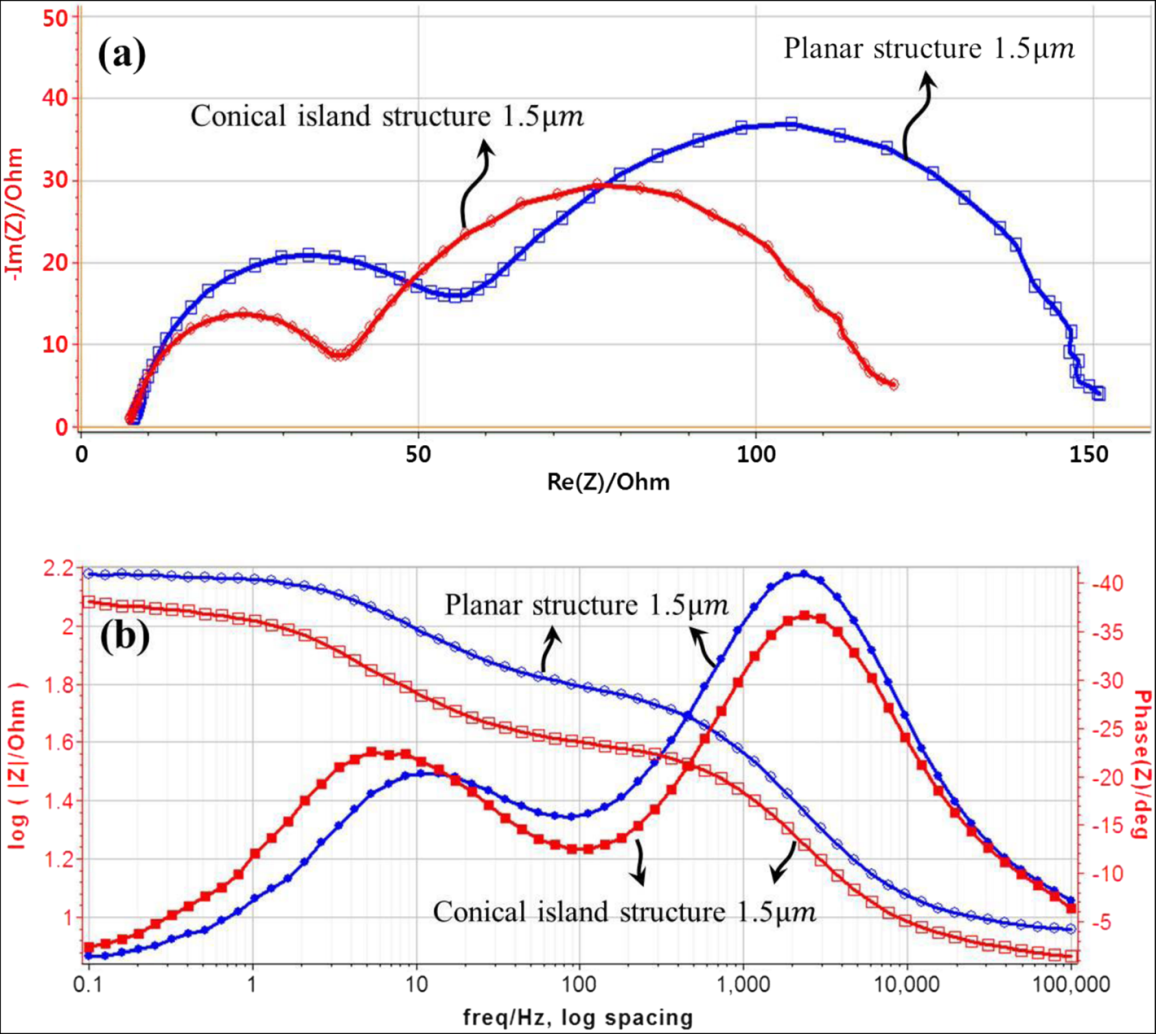
**EIS results of DSCs based on planar and conical island structures measured at open-circuit condition under 1.0-sun illumination. (a)** Nyquist plots and **(b)** Bode plots.

Figure 7 shows EIS results of DSCs with planar and conical island structures. The Nyquist plots in Figure 7a show that DSCs based on conical island structures have a lower chemical resistance than DSCs based on planar structures. Lower chemical resistance is closely related to better efficiency in DSCs. These results exactly coincide with the *J*-*V* characteristics in Figure 6 and Table 1.

Chemical resistances in Figure 7a include the charge transfer resistance (*R*_Pt/electrolyte_) at the interface between the Pt counter electrode and electrolyte, charge transfer resistance (*R*_TiO2/electrolyte_) at the interface between the TiO_2_ photoelectrode and electrolyte, and charge transport resistance (*R*_electrolyte_) in the electrolyte. DSCs based on the conical island structure have lower chemical resistance due to remarkably decreased *R*_Pt/electrolyte_. The reason for this decrease in *R*_Pt/electrolyte_ is not clear. It seems that the conical island may be a better structure for transferring electrons to the counter electrode than the planar structure, since the *J*_sc_ of the conical island structure is higher than that of the planar structure.

Figure 7b shows Bode plots of DSCs based on planar and conical island structures. Usually, Bode plots of DSCs have three main phase (θ) peaks, according to the frequency range. The high-frequency (1 kHz to 100 kHz) region is attributed to charge transfer at the interface between the counter cathode and electrolyte. The medium-frequency (1 Hz to 1 kHz) region is the response of charge transfer at the interface between the TiO_2_ photoelectrode and electrolyte. The low-frequency (1 mHz to 1 Hz) region reflects charge transport associated with the Nernstian diffusion process in the electrolyte. If the frequency at the phase peaks can be determined, the electronic lifetimes at each interface and in the electrolyte can be calculated by just inversing the peak frequencies. Only two phase peaks, in the high- and medium-frequency regions, could be found in Figure 7b. The phase peak in the low-frequency region was not observed because the acquisition time was too short to obtain the diffusion peak.

In the high-frequency region, frequencies at the phase peaks are approximately 2.1 kHz and approximately 2.4 kHz for DSCs based on planar and conical island structures, respectively; therefore, the electronic lifetimes are 0.476 and 0.417 ms, respectively. On the other hand, in the medium-frequency region, the frequencies at phase peaks are 10 and 5.5 Hz for DSCs with planar and conical island structures; thus, the electronic lifetimes are 0.1 and 0.18 s, respectively. The difference of electronic lifetimes in the high-frequency region is negligible; however, in the medium-frequency region, the electronic lifetime of DSCs based on the conical island structure is obviously longer than that of DSCs based on the planar structure. These results indicate that conical islands composed of TiO_2_ nanotubes are more effective for charge transfer at the interface between the TiO_2_ photoelectrode and electrolyte.

**Figure 8:**
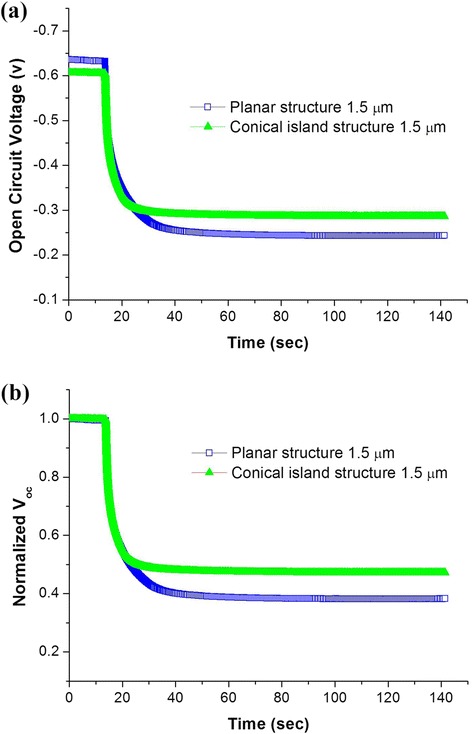
**Open-circuit voltage decay measurements of DSCs based on planar and conical island structures. (a)** Raw data of open-circuit voltage decay and **(b)** normalized open-circuit voltage decay.

Figure 8 shows the raw data and normalized values of open-circuit voltage (*V*_oc_) decay measurements for DSCs based on planar and conical island structures. To analyze DSC *V*_oc_ decay behaviors, light at AM 1.5G was supplied for approximately 20 s, and *V*_oc_ decays were measured by abruptly stopping the supply of illumination. When the illumination on DSCs is stopped, charge recombination at the interface of the TiO_2_ photoelectrode or counter electrode gives rise to the decrease of *V*_oc_. The slower the *V*_oc_ decay is, the higher the electronic lifetime is. This test revealed that the *V*_oc_ decay of DSCs based on the conical island structure was slower than that of DSCs based on the planar structure; therefore, the electronic lifetime of DSCs based on the conical island structure is higher than that of DSCs based on the planar structure. This result is closely correlated to the result of Bode plots in Figure 7b.

## Conclusions

Ti conical islands were fabricated by coating photoresists on Ti foils, stacking dot-patterned photomasks on the photoresists, illuminating with UV light, etching the surface with the RIE method, and stripping the photoresist. When the produced Ti conical islands were anodized, conical islands composed of TiO_2_ nanotubes were successfully fabricated. The conical islands composed of TiO_2_ nanotubes were then employed as photoelectrodes of DSCs. The *J*-*V* characteristics of DSCs based on the conical island structures were compared with those of DSCs based on the planar structures covered with TiO_2_ nanotubes. The *J*_sc_ and the efficiency of DSCs based on the conical island structures were higher than those of DSCs based on the planar structures. The efficiency of the DSCs using conical island structures reached 1.87%. The charge transfer resistance at the counter cathode of DSCs based on the conical island structures was remarkably reduced compared to that of DSCs based on the planar structures. The electron lifetime at the interface between the photoelectrode and electrolyte was longer for DSCs based on the conical island structure than that for DSCs based on the planar structure. The conical island structure effectively enhanced the performance of DSCs based on TiO_2_ nanotube arrays. The relatively low efficiency of DSCs based on the conical island structure compared with that of typical DSCs can be overcome by controlling the microstructure of the conical island, dye, electrolyte, surface treatment of TiCl_4_, and so on.
